# Beneficial Insect Borders Provide Northern Bobwhite Brood Habitat

**DOI:** 10.1371/journal.pone.0083815

**Published:** 2013-12-23

**Authors:** Christopher E. Moorman, Charles J. Plush, David B. Orr, Chris Reberg-Horton

**Affiliations:** 1 Fisheries, Wildlife, and Conservation Biology Program, North Carolina State University, Raleigh, North Carolina, United States of America; 2 Department of Entomology, North Carolina State University, Raleigh, North Carolina, United States of America; 3 Department of Crop Sciences, North Carolina State University, Raleigh, North Carolina, United States of America; University of Alberta, Canada

## Abstract

Strips of fallow vegetation along cropland borders are an effective strategy for providing brood habitat for declining populations of upland game birds (Order: Galliformes), including northern bobwhite (*Colinus virginianus*), but fallow borders lack nectar-producing vegetation needed to sustain many beneficial insect populations (e.g., crop pest predators, parasitoids, and pollinator species). Planted borders that contain mixes of prairie flowers and grasses are designed to harbor more diverse arthropod communities, but the relative value of these borders as brood habitat is unknown. We used groups of six human-imprinted northern bobwhite chicks as a bioassay for comparing four different border treatments (planted native grass and prairie flowers, planted prairie flowers only, fallow vegetation, or mowed vegetation) as northern bobwhite brood habitat from June-August 2009 and 2010. All field border treatments were established around nine organic crop fields. Groups of chicks were led through borders for 30-min foraging trials and immediately euthanized, and eaten arthropods in crops and gizzards were measured to calculate a foraging rate for each border treatment. We estimated arthropod prey availability within each border treatment using a modified blower-vac to sample arthropods at the vegetation strata where chicks foraged. Foraging rate did not differ among border treatments in 2009 or 2010. Total arthropod prey densities calculated from blower-vac samples did not differ among border treatments in 2009 or 2010. Our results showed plant communities established to attract beneficial insects should maximize the biodiversity potential of field border establishment by providing habitat for beneficial insects and young upland game birds.

## Introduction

Uncultivated field margins (hereafter, field borders) are an effective practice for providing multiple ecological services within agricultural landscapes. Field borders aid in erosion control, improve water quality near riparian areas, and provide wildlife habitat [1,2]. The establishment of field borders is both simple and cost-effective for landowners, requiring only that natural vegetation be allowed to grow along the interface of crop and field edge where crop productivity is inherently poor. Borders also are effective in straightening oddly shaped fields, making it easier for producers to efficiently operate machinery during agricultural activities.

Field borders have been recognized as a possible venue for promoting natural crop pest enemies and pollinator species (collectively referred to as beneficial insects), but borders composed of fallow vegetation offer poor habitat for beneficial insect populations, largely because of the lack of nectar-producing plants within the borders [3]. Predators and parasitoids of crop pests require diverse habitats that provide abundant pollen and nectar sources and differential microhabitats used as nesting and over-wintering sites throughout the year [4]. Sugar obtained from pollen and nectar is essential for beneficial insect reproduction, and serves as an alternate food source for predator species in times when prey species are less abundant [5]. The young of parasitoid wasps feed on their host, whereas adults of these species often rely exclusively on nectar and pollen to meet caloric demands. Vegetation communities in agricultural systems lacking in nectar and pollen greatly reduces predator and parasitoid species’ ability to control pest species that are abundant in monoculture crops [4,6]. Fallow field borders established to conserve northern bobwhite (*Colinus virginianus*) in Georgia, USA, were unsuitable for increasing beneficial insect populations [3]. However, increases in desired arthropod communities are possible through habitat manipulation that promotes specific vegetation [7,8].

Populations of upland game birds (Order: Galliformes) are declining globally, largely due to the loss or degradation of early-successional vegetation types essential to life cycle functioning [9,10]. Cooperators have initiated programs that encourage establishment of fallow field borders and other set-aside lands on agricultural lands in an effort to increase upland game bird populations [11]. Vegetation structure in field borders can provide the closed overhead canopy and bare ground microhabitat selected by bobwhite and other upland game birds for brood-rearing, foraging, and movement [12]. Annual and perennial weeds attract insects vital to game bird chick development [13]. Field borders have been a widely accepted practice in the eastern United States because landowners suffer minimal losses in agricultural productivity and local northern bobwhite populations have been shown to increase where field borders have been established [2]. Establishing strips of non-crop vegetation through the center of agricultural fields in Europe also has benefited gray partridge (*Perdix perdix*) [14].

We compared the value of field borders planted for promoting beneficial insects to traditional fallow field borders as brood cover for northern bobwhite (hereafter, bobwhite), an upland game bird that has declined precipitously across most of its range [15]. Lack of available brood cover that provides access to arthropod food sources can limit bobwhite and other game bird populations. We investigated how beneficial insect habitats and the associated plant community composition and structure affected bobwhite chick foraging success relative to fallow borders or the mowed areas common across much of the bobwhite’s range. Our objective was to compare the value of beneficial insect habitats as brood habitat for northern bobwhite to other border types. We: 1) compared foraging rates of human-imprinted bobwhite chicks among different field border types; and 2) compared the abundance and diversity of available arthropod food sources among border types. 

## Materials and Methods

### Ethics Statement

All research, including care, handling, and euthanasia of bobwhite chicks, was approved by the North Carolina State University Institutional Animal Care and Use Committee under protocol 09-052-O. All efforts were made to minimize suffering of northern bobwhite chicks during the study. The work at the Center for Environmental Farming Systems (CEFS) was approved by both the North Carolina Department of Agriculture and the North Carolina Agricultural Experiment Station.  

### Study Area and Field Border Establishment

Our study was conducted at the CEFS Organic Research Unit (ORU) in the upper coastal-plain physiographical region of North Carolina, USA during 2009-2010. The ORU consisted of nine organic crop fields ranging from 1.6 to 4 ha in size. All agricultural activities followed United States Department of Agriculture organic crop production guidelines. Four randomly assigned border habitat treatments were established in spring 2008 around each of the nine crop fields yielding a total of 36 experimental units. Field borders were established along crop field margins and were bordered by other crop fields, shrubland, forest, railroad, or farm buildings. Each habitat treatment was approximately 91.4 m long by 9.1 m wide, for a total of 0.33 ha of experimental habitat in each field. The four border habitat treatments were: 1) planted native-warm season grasses (NWSG) and native prairie flowers (hereafter NWSG/Flowers); 2) planted native prairie flowers only (hereafter Flowers Only); 3) fallow, unmanaged vegetation (hereafter Fallow); 4) volunteer grasses and herbaceous vegetation mowed two to three times per month (hereafter Mowed). The NWSG species planted were Indiangrass (*Sorghastrum nutans*) and little bluestem (*Schizachyrium scoparium*). Native prairie flower species planted in NWSG/Flowers and Flowers Only borders were lance-leaved coreopsis (*Coreopsis lanceolata*), purple coneflower (*Echinacea purpurea*), black-eyed susan (*Rudbeckia hirta*), butterfly milkweed (*Asclepias tuberosa*), common milkweed (*Asclepias syriaca*), swamp sunflower (*Helianthus angustifolius*), heath aster (*Symphyotrichum pilosum*), and showy goldenrod (*Solidago speciosa*). We selected species planted in the NWSG/Flowers and Flowers Only borders because their seeds are readily available for purchase, they were native to the United States, they were adaptable to local soils and climate, and they have demonstrated value for beneficial insects [16]. The mix of flower species bloom at different dates, providing nectar sources throughout the growing season. The fallow border represented traditional field borders established for wildlife habitat, and we included the mowed treatment as a comparison to the management of cropland margins commonly implemented on farm landscapes across the United States.

Planted field borders were established by disking the treatment area and broadcasting the seed mix over the tilled soil using a manually powered seed spreader. A culti-packer was run over the treatment area to ensure good seed-to-soil contact following broadcasting. Once vegetation in planted field borders reached approximately 30 cm high, it was mowed at a height of 15 cm, 5-6 times throughout the 2008 growing season to reduce weed competition and to promote sound stand establishment. We performed no further management activities on any of the planted field border treatments following the 2008 growing season. Fallow field borders were tilled in fall 2007, and left to return to natural vegetation for the duration of the study. Fallow field border vegetation consisted of a mix of grasses, primarily non-native bermudagrass (*Cynodon dactylon*) and non-native crabgrass (*Digitaria ciliaris*), and commonly occurring herbaceous species, such as horseweed (*Conyza canadensis*), dogfennel (*Eupatorium capillifolium*), heath aster (*Symphyotrichum pilosum*), pigweed (*Amaranthus* spp.), and non-native sicklepod (*Senna obtusifolia*). Baccharis (*Baccharis halimifolia*), a woody species, also became prevalent within fallow borders two years following border establishment. A more comprehensive description of vegetation composition and structure in each border type is provided elsewhere [17]. 

### Northern Bobwhite Foraging Trials

The use of human-imprinted chicks in studying food preferences and foraging habits is well documented for bobwhite and other game bird species [18-20]. We used 10-12 day old human-imprinted bobwhite chicks to conduct three foraging trials each year from June-August in 2009 and 2010. We scheduled foraging trials to coincide with the primary brooding periods of wild bobwhites in the southeastern United States.

We purchased 100-150 pen-strain bobwhite eggs from a local breeder prior to each trial, and upon delivery placed them in a commercial incubator (Jamesway Incubator Model 252, Butler Manufacturing, Fort Atkinson, WI). We assumed pen-strain chicks would forage equally to genetically wild bobwhite [20]. We incubated eggs at temperature and humidity levels necessary for proper chick development, and eggs were rotated automatically four times daily. We placed eggs in hatching trays after 21 days of incubation and transported them to a commercial hatcher. A tape recorder inside the hatcher played a recording of a three-note whistle call and the researcher’s voice continuously at 1-min intervals. Chicks are able to hear within the egg at 21 days of incubation, thus playing of the recordings began the human imprinting process [18]. We placed hatched chicks in a towel held by a researcher, and transported each to the rearing facility.

We constantly talked to the birds, whistled to them, congregated them under our hands, and held them next to our bodies during the initial 24-h period after hatching (hereafter Day 0). We fed chicks wild arthropods captured nearby on Day 1, and groups of 30-40 chicks were designated by painting different colors of nail polish on their heads on Day 2. We exercised chicks for at least 1 h, twice daily in designated groups in nearby lawns, fields, or weedy areas beginning on Day 3. The exercise sessions were intended to expose the chicks to vegetation similar to that used in the foraging trials and to familiarize chicks with arthropod foraging. Chicks that did not respond to the observers when called or frequently produced “lost calls” were considered unsuccessfully imprinted and were immediately removed from the study. We kept chicks in an imprinting ring (i.e., cardboard ring 3 m diameter and 0.3 m high with cedar chip bedding and a brooder lamp) and provided them with commercial game bird starter food (Game Bird Startena, Purina ®) and water *ad libitum* between exercise sessions. A recording of the three-note whistle and the researchers’ voice was left playing to provide assurance to the chicks during any period when researchers were not with the chicks. Exercise sessions continued daily until the chicks were 10-12 days of age when they were transported to the site of foraging trials.

We transported chicks via automobile to CEFS the morning prior to foraging trials. Chicks were restricted of any arthropod foods 18 h prior to the trials, and restricted of all foods 4 h before trials to ensure that crops and gizzards were flushed completely of any arthropod fragments and to encourage foraging. We performed foraging trials over a 2-day period during which all borders around three of the crop fields were sampled, for a total of 12 borders sampled per trial. All borders around each of the nine fields were sampled once each year. We conducted trials only on dry days between 0900 and 1200 hours to ensure all vegetation was dry and insect movement was not impeded by moisture. At least four observers were present on the day of each trial. A trial was begun when a pair of observers released a group of six chicks at the end of a specified border. One observer stood behind the brood of chicks while the other stood in the middle of the border 15 m in front of the brood. The trailing observer kept a constant count of the brood and ensured that chicks did not forage outside of the border. The distant observer would whistle at 1-min intervals to ensure the brood would continue foraging on the correct path. The observer roles would reverse whenever the brood reached the distant observer, and the distant observer would begin trailing the brood while the original trailer would walk outside the border and reestablish a position 15-20 m from the brood further down the border. We allowed chicks to forage freely within the border boundaries, with no contact or interference from observers. We conducted trials for exactly 30 min and immediately collected and euthanized chicks via cervical dislocation at the end of each trial. One chick was excluded in 2009 and 10 chicks were excluded in 2010 because they were not captured immediately after the 30-min time limit. We placed chicks in plastic bags and stored them in portable coolers containing ice and later in the day stored them in a freezer at 17.8° C. 

### Crop and Gizzard Analysis

We placed frozen chicks in a refrigerator to thaw for at least 12 h. We extracted the crop, esophagus, and gizzard from each thawed chick, and stored the digestive organs in a 70% ethyl alcohol solution. Four chicks from 2010 were excluded from analysis because their digestive organs were damaged during extraction. We opened the digestive organs with a scalpel, and rinsed their contents with an ethyl alcohol solution into a petri dish. We identified whole arthropods to family using a 30x dissecting microscope and measured the length and width of each arthropod to the nearest 0.01 mm using digital calipers. We also searched the crop and gizzard contents for diagnostic arthropod fragments (i.e., mandibles, tibias, wings). Counting and identification of arthropods observed as diagnostic fragments followed protocols outlined in [19]. We identified fragments to family when possible; order was recorded otherwise. We recorded lengths and widths of diagnostic fragments as well. 

### Arthropod Sampling

A number of sampling methodologies have been developed for assessing arthropod abundance and diversity, including sweep nets, pitfall traps, and drift fences. Relative abundance values obtained using these methods likely are biased, because the methods may fail to sample from the arthropod communities most readily available to bobwhite chicks [19]. For example, sweep netting may only capture insects occupying the upper half of the vegetation strata, and pitfall traps may not capture insects that have a limited movement range. We minimized these biases by using an arthropod sampling technique that is indiscriminate of vegetation structure or species characteristics. Our technique resulted in sampling arthropods from the vegetation strata where bobwhite chicks forage.

We used a modified, gas-powered blower-vacuum as a vacuum sampler similar to that described in [21] to sample arthropods in the different border types. Our sampling approach was designed to quantify arthropods available to bobwhite chicks and was not intended to measure beneficial insect response to the four border types. We conducted arthropod sampling during the day between 0900 and 1200 within 3 days following a foraging trial to capture arthropods representative of the time when foraging trials were conducted. We collected arthropods in each field border using the vacuum sampler along three, 3.05-m transects running perpendicular to the length of each border. Transect lines were distributed randomly across the length of each border, and were marked 2 days prior to arthropod sampling. We calculated arthropod density by multiplying the length of each transect line (3.05 m) by the vacuum sampler inlet width (0.13 m) (Density= # arthropods/ 0.38 m^2^). We held the vacuum sampler inlet as far ahead of the operator as possible to capture insects before they flushed. The inlet of the suction sampler was held approximately 15 cm from the soil surface to collect arthropods from the lower levels of the vegetation strata and soil surface to approximate the zone where quail chicks feed. Collection bags were held in a cooler with ice until transferred to a freezer for storage at 17.8° C. 

We dried bag contents at least 1 week prior to analysis. Contents were emptied on a white sorting tray, and arthropods were sorted with tweezers and a hand lens. We counted all arthropods and identified them to family. 

### Statistical Analyses

We analyzed differences in northern bobwhite foraging rates, arthropod availability, and vegetation characteristics among border treatments separately for each year, because vegetation structure and composition changed drastically over the 2 years following establishment. We used allometric equations to calculate the estimated live weight of each arthropod consumed by each chick during foraging trials [22]. We summed the estimated live weights of each arthropod consumed by each chick to calculate a foraging rate (g of arthropods consumed/chick/30 min). We performed an analysis of variance (ANOVA) using Proc MIXED (SAS Institute Inc., Cary, NC) to test for differences in foraging rates among year and field border treatments. We also performed ANOVAs using Proc MIXED to test for differences in the mass of each taxonomic order and family consumed by chicks among border treatments. In all models, we treated border treatment as a fixed effect, and field as a random effect. We also included chick nested within the interaction between field and border treatment as a random repeated measures effect to account for variability among individual chicks. We used Tukey-Kramer adjustments in pairwise comparisons to evaluate differences in foraging rates, weight of taxonomic order consumed, and weight of taxonomic family consumed relative to border treatment. We only reported the mean mass of arthropods consumed per chick to taxonomic orders and families that made up a significant portion of bobwhite chicks’ diet. We excluded certain taxonomic orders and families, such as Diptera and Neuroptera, from individual comparisons among field border treatments because the amount of mass consumed within those taxonomic groups was too small to make statistical comparisons.

We performed individual ANOVAs using Proc MIXED to compare overall arthropod prey abundance and abundance of arthropods in each taxonomic order and family among field border treatments. We included border treatment as a fixed effect and field as a random effect in all models. We used Tukey-Kramer adjustments in pairwise comparisons to compare the total number of arthropods and the number of arthropods within each taxonomic order and family among border treatments. We also calculated a Shannon-Weiner diversity index value (H’) for each border treatment in each year to assess arthropod diversity in border treatments. The index was scored on a 0-4 scale, with higher values suggesting greater arthropod diversity. Significance was accepted at P ≤ 0.05 for all statistical tests.

## Results

### Northern Bobwhite Foraging Trials

We led 432 bobwhite chicks through foraging trials during 2009-2010. We excluded one chick from analysis in 2009, and 14 chicks in 2010. We identified 2,656 arthropods consumed by chicks in 2009, and 2,267 arthropods consumed in 2010. Arthropods in the taxonomic orders Coleoptera, Hemiptera, and Hymenoptera made up the greatest percentage of chicks’ diets in all border treatments. Carabid beetles (Family: Carabidae) and darkling beetles (Family: Tenebrionidae) comprised the greatest percentage of coleopteran families consumed by chicks in both years. Stinkbug nymphs (Family: Pentatomidae) and damselbugs (Family: Nabidae) were consumed in the greatest proportions among hemipteran families. The majority of hymenopterans consumed by chicks in all border treatments were ants (Family: Formicidae) (Table 1).

**Table 1:** Percentage of arthropods consumed by northern bobwhite chicks in four field border treatments by taxonomic order and most frequently consumed taxonomic family (June to August 2009 and 2010).

	NWSG/Flowers	Flowers Only	Fallow	Mowed
Araneae	13.43	15.46	11.33	11.84
Chilopoda	1.48	0.52	0.16	0.29
Coleoptera	37.24	33.36	30.42	27.89
Carabidae	14.13	12.59	9.06	8.12
Curculionidae	5.39	6.63	6.07	4.30
Tenebrionidae	10.77	5.38	5.42	8.21
Dermaptera	0.00	0.07	0.00	0.00
Diptera	0.31	0.15	0.16	0.48
Gastropoda	0.00	0.00	0.16	0.29
Hemiptera	20.45	22.90	27.51	31.61
Cicadellidae	3.51	2.28	1.86	3.72
Nabidae	4.29	2.36	4.94	3.63
Pentatomidae	4.37	5.52	7.85	4.87
Hymenoptera	21.55	19.44	19.58	19.48
Formicidae	20.37	17.89	18.28	18.43
Lep Larvae	1.80	3.24	6.31	5.73
Orthoptera	3.75	4.12	3.56	2.10
Acrididae	3.75	3.90	3.48	2.01
	n^a^ =1,282	n=1,359	n=1,236	n=1,046

^a^ n is the number of arthropods consumed by all chicks in each field border treatment in 2009 and 2010.

**Figure 1:**
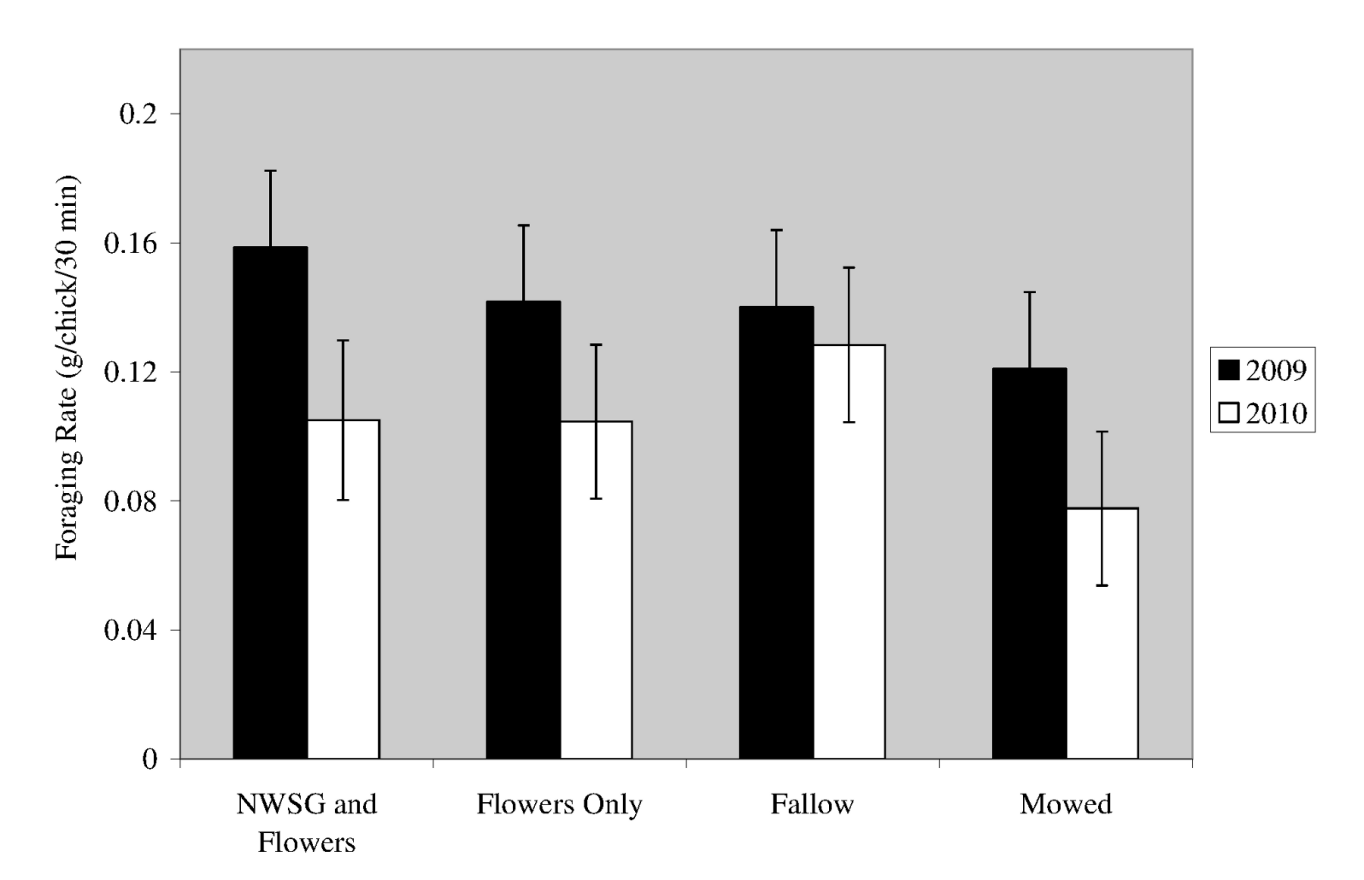
Estimated least-square mean (± standard error) foraging rates for northern bobwhite chicks within field border treatments. Foraging rates were collected in North Carolina in 2009 and 2010. Foraging rates are reported as g of arthropods consumed/chick/30 min. Least-square mean estimates and SE were derived from MIXED models.

We did not detect a difference in foraging rate among border treatments in 2009 (*F*_*3,201,=*_ 0.63, *P*=0.60), or in 2010 (*F*_*3,188*_ =2.17, *P*=0.60) (Figure 1). We excluded one Fallow border replicate from analysis in 2010, because the border was treated with methyl bromide to eradicate tropical spiderwort (*Commelina benghalensis*), a federally listed noxious weed. The successional age and species composition of the vegetation within this border were not comparable to other Fallow border replicates in 2010, because of the chemical treatment.

Lepidoptera larvae (caterpillars), Orthoptera, and coleopteran species contributed the greatest mean mass of arthropods consumed per chick among taxonomic orders in all field border treatments. Ants (Family: Formicidae) and hemipteran species contributed little to the total amount of biomass consumed by each chick, although they comprised a large percentage of the prey items in chicks’ diets. Grasshoppers (Family: Acrididae) had the greatest mean weight within Orthoptera, and carabid beetles had the greatest mean weight within Coleoptera (Table 2).

**Table 2:** Estimated least-square mean ± SE mass (mg) of most important taxonomic orders and families of arthropods consumed per northern bobwhite chick over 30-minute foraging trials in four field border treatments (June to August 2009 and 2010).

	NWSG/Flowers**^*a*^**	Flowers Only	Fallow	Mowed
2009				
Araneae	7.5 ± 2.1^B^	8.5 ± 2.1^B^	14.7 ± 2.1^A^	11.8 ± 2.1^AB^
All Coleoptera	29.3 ± 5.0^A^	16.6 ± 4.8^B^	17.2 ± 4.7 ^B^	14.1 ± 4.7 ^B^
Carabidae	84.5 ± 11.8^A^	63.7 ± 11.8^AB^	54.2 ± 11.8^AB^	40.3 ± 12.5^B^
Curculionidae	5.6 ± 2.0	4.8 ± 1.6	5.8 ± 1.4	4.8 ± 1.5
Tenebrionidae	23.5 ± 3.6^A^	11.6 ± 3.8^B^	17.8 ± 4.9^AB^	16.0 ± 3.8^AB^
All Hemiptera	8.4 ± 1.7	8.0 ± 1.7	9.5 ± 1.6	7.5 ± 1.7
Cicadellidae	8.0 ± 3.6	10.1 ± 3.8	6.2 ± 4.5	12.2 ± 3.6
Nabidae	8.8 ± 1.3^AB^	8.7 ± 1.4^AB^	10.3 ± 1.3^A^	5.9 ± 1.4^B^
Pentatomidae	9.2 ± 8.5	9.1 ± 5.4	17.7 ± 4.0	11.0 ± 6.0
All Hymenoptera	2.7 ± 1.4	1.5 ± 1.4	1.5 ± 1.5	2.1 ± 1.4
Formicidae	2.8 ± 1.4	1.4 ± 1.4	1.4 ± 1.5	2.4 ± 1.5
Lep Larvae	65.4 ± 43.2	60.2 ± 38.7	55.0 ± 29.0	82.1 ± 43.1
All Orthoptera	53.3 ± 44.4^B^	85.4 ± 44.6^AB^	65.0 ± 44.5^AB^	195.0 ± 47.2^A^
Acrididae	53.3 ± 44.0^B^	117.9 ± 49.8^AB^	42.9 ± 46.6^B^	218.0 ± 49.8^A^
2010				
Araneae	5.0 ± 2.1	4.9 ± 2.1	4.9 ± 2.1	3.1 ± 2.2
All Coleoptera	12.0 ± 5.1	10.4 ± 5.0	14.4 ± 5.0	6.4 ± 5.2
Carabidae	30.5 ± 14.4^AB^	29.6 ± 11.8^AB^	53.7 ± 12.5^A^	15.9 ± 12.5^B^
Curculionidae	9.5 ± 1.5^A^	8.1 ± 1.4^AB^	6.2 ± 1.4^AB^	4.7 ± 1.6^B^
Tenebrionidae	16.8 ± 4.9	14.4 ± 4.1	12.2 ± 6.3	16.0 ± 4.9
All Hemiptera	11.2 ± 1.9^A^	11.2 ± 1.8^A^	10.3 ± 1.8^A^	6.7 ± 1.8^B^
Cicadellidae	5.3 ± 4.1	6.4 ± 5.0	5.6 ± 5.8	4.6 ± 3.8
Nabidae	6.6 ± 1.5	4.5 ± 1.6	5.8 ± 1.8	5.6 ± 1.6
Pentatomidae	27.7 ± 4.2^A^	23.2 ± 4.0^A^	18.7 ± 4.0^B^	10.0 ± 4.2^B^
All Hymenoptera	9.8 ± 1.4	8.1 ± 1.5	8.9 ± 1.5	7.6 ± 1.5
Formicidae	9.1 ± 1.5	9.5 ± 1.4	9.9 ± 1.4	7.9 ± 1.4
Lep Larvae	19.9 ± 30.7^B^	45.0 ± 30.7^AB^	121.0 ± 38.7^A^	116.7 ± 49.7^A^
All Orthoptera	55.6 ± 50.4	48.0 ± 47.2	102.9 ± 54.5	44.3 ± 66.7
Acrididae	55.2 ± 49.8	47.7 ± 46.7	102.5 ± 53.8	43.7 ± 65.8

^a^ Within rows, means followed by different letters were statistically different (*P*<0.05).

Chicks foraging in NWSG/Flowers borders in 2009 consumed over 41% greater mass of Coleoptera than in all other border treatments. Mean mass of carabid beetles consumed was approximately 52% lower in Mowed borders compared to NWSG/Flowers borders, but did not differ among NWSG/Flowers, Flowers Only, and Fallow borders. Mass of Hemiptera consumed per chick did not differ among border treatments in 2009, but was over 34% lower in Mowed borders in 2010 compared to all other border treatments. Chicks foraging in Mowed borders in 2009 consumed over 75% greater mass of grasshoppers compared to NWSG/Flowers and Flowers Only borders. Mass of spiders (Order: Araneae) consumed was over 42% greater in Fallow borders than in NWSG/Flowers and Flowers Only borders in 2009 (Table 2). 

Mass of Araneae, Coleoptera, and Hymenoptera consumed in 2010 did not differ among border treatments. However, mass of carabid beetles consumed was approximately three times greater in Fallow borders compared to Mowed borders. Chicks consumed a greater mass of Pentatomidae in NWSG/Flowers and Flowers Only borders in 2010 than in Fallow and Mowed borders. Chicks ate 84% greater mass of Lepidoptera larvae in Fallow borders in 2010 than in NWSG/Flowers borders (Table 2). 

### Arthropod Sampling

We collected 212 blower-vacuum samples from 2009-2010. Total arthropod densities ranged from 33.67-42.26 arthropods/0.38 m^2^ in 2009, and 36.07-43.63 arthropods/0.38 m^2^ in 2010. The Shannon-Weiner diversity index (H’), in both 2009 and 2010, was greatest in Fallow borders and lowest in Mowed borders (Table 3). Total arthropod density was not different among border treatments in 2009 or 2010. Araneae density in 2009 was over 41% greater in NWSG/Flowers and Flowers Only borders than in Mowed borders, and Hemiptera density was over 38% less in NWSG/Flowers borders than in Fallow and Mowed borders. Cicadellidae density was approximately two times greater in Fallow and Mowed borders than in NWSG/Flowers and Flowers Only borders. Mean density of Coleoptera, Hymenoptera, and Lepidoptera larvae were not different among border treatments in 2009. Orthoptera density was nearly two times greater in Flowers Only borders compared to NWSG/Flowers and Mowed borders in 2009 (Table 3).

**Table 3:** Estimated least-square mean ± SE number of arthropods per 0.38 m^2^ of most important taxonomic orders and families in four field border treatments (June to August 2009 and 2010).

	NWSG/Flowers**^*a*^**	Flowers Only	Fallow	Mowed
2009				
Araneae	8.9 ± 1.4^A^	9.0**^*a*^** ± 1.4^A^	7.4 ± 1.4^AB^	5.2 ± 1.4^B^
All Coleoptera	5.2 ± 1.0	5.7 ± 1.0	5.4 ± 1.0	4.3 ± 1.0
Carabidae	1.7 ± 0.7	2.7 ± 0.7	1.3 ± 0.7	1.5 ± 0.7
Curculionidae	0.5 ± 0.2	0.6 ± 0.2	0.5 ± 0.2	0.2 ± 0.2
Tenebrionidae	0.3 ± 0.1	0.3 ± 0.1	0.4 ± 0.1	0.3 ± 0.1
All Hemiptera	8.1 ± 2.4^B^	12.4 ± 2.4^AB^	17.4 ± 2.4^A^	13.3 ± 2.4^A^
Cicadellidae	2.4 ± 1.5^B^	3.9 ± 1.5^B^	6.6 ± 1.5^A^	6.8 ± 1.5^A^
Nabidae	0.5 ± 0.3	0.4 ± 0.3	1.2 ± 0.3	0.5 ± 0.3
Pentatomidae	0.0 ± 0.1	0.1 ± 0.1	0.1 ± 0.1	0.1 ± 0.1
All Hymenoptera	3.3 ± 0.9	4.2 ± 0.9	5.1 ± 0.9	2.9 ± 0.9
Formicidae	1.8 ± 0.9	2.6 ± 0.9	2.1 ± 0.9	1.6 ± 0.9
Lep Larvae	0.1 ± 0.1	0.0 ± 0.1	0.1 ± 0.1	0.1 ± 0.1
All Orthoptera	2.6 ± 0.8^BC^	4.3 ± 0.8^A^	3.4 ± 0.8^AB^	1.7 ± 0.8^C^
Acrididae	0.9 ± 0.4^B^	1.0 ± 0.4^B^	2.3 ± 0.4^A^	1.4 ± 0.4^B^
All Arthropods	33.9 ± 4.9	38.6 ± 4.9	42.3 ± 4.9	33.7 ± 4.9
H’^b^	2.9	3.0	3.1	2.8
	n^c^=27	n=27	n=25	n=27
2010				
Araneae	11.1 ± 1.2^A^	8.0 ± 1.2^B^	8.0 ± 1.2^B^	7.0 ± 1.2^B^
All Coleoptera	7.3 ± 1.5^A^	5.2 ± 1.5^AB^	5.3 ± 1.5^AB^	4.0 ± 1.5^B^
Carabidae	2.9 ± 0.8	1.5 ± 0.8	2.0 ± 0.8	1.5 ± 0.8
Curculionidae	1.3 ± 0.3^A^	1.4 ± 0.3^A^	0.9 ± 0.3^AB^	0.4 ± 0.3^B^
Tenebrionidae	0.0 ± 0.1	0.2 ± 0.1	0.0 ± 0.1	0.0 ± 0.1
All Hemiptera	5.6 ± 1.5^B^	5.3 ± 1.5^B^	7.2 ± 1.5^AB^	10.7 ± 1.5^A^
Cicadellidae	2.2 ± 1.0^B^	2.4 ± 1.0^B^	2.6 ± 1.0^B^	5.9 ± 1.0^A^
Nabidae	0.6 ± 0.2	0.1 ± 0.2	0.1 ± 0.2	0.2 ± 0.2
Pentatomidae	0.1 ± 0.1	0.1 ± 0.1	0.1 ± 0.1	0.2 ± 0.1
All Hymenoptera	6.9 ± 1.5^A^	7.2 ± 1.5^A^	9.2 ± 1.5^A^	3.6 ± 1.5^B^
Formicidae	3.2 ± 1.3^AB^	3.2 ± 1.3^AB^	5.6 ± 1.3^A^	1.8 ± 1.3^B^
Lep Larvae	0.2 ± 0.2^B^	0.3 ± 0.2^AB^	0.5 ± 0.2^AB^	0.7 ± 0.2^A^
All Orthoptera	1.4 ± 0.3	1.3 ± 0.3	1.4 ± 0.3	1.3 ± 0.3
Acrididae	1.1 ± 0.3	1.0 ± 0.3	0.8 ± 0.3	1.0 ± 0.3
All Arthropods	40.2 ± 5.4	36.1 ± 5.4	42.2 ± 5.4	43.6 ± 5.4
H’	2.5	2.5	2.6	2.2
	n=27	n=27	n=25	n=27

^a^ Within rows, means followed by different letters were statistically different (*P*<0.05).

^b^ H’=Shannon-Weiner Diversity Index.

^c^ n is the number of samples collected in each border treatment.

Araneae density in 2010 was over 27% greater in NWSG/Flowers borders than all other border treatments, and Coleoptera density was approximately two times greater in NWSG/Flowers borders than in Mowed borders. Weevil (Family: Curculionidae) density was over 65% greater in NWSG/Flowers and Flowers Only borders than in Mowed borders. Mean density of Hemiptera was approximately two times greater in Mowed borders than in NWSG/Flowers and Flowers Only borders. Density of leafhoppers (Family: Cicadellidae) was over 56% greater in Mowed borders than in all other border treatments. There were over two times less Hymenoptera in Mowed borders in 2010 than in all other border treatments, but there was no difference in the density of Orthoptera among border treatments. Density of Lepidoptera larvae was over 75% lower in NWSG/Flowers borders compared to Mowed borders in 2010 (Table 3).

## Discussion

Plant communities established to attract beneficial insects may maximize the biodiversity value of field borders by conserving beneficial insects and providing brood habitat for northern bobwhite, and possibly other upland game birds. Establishment of fallow field borders on only 2-3% of row crop area increased bobwhite abundance on farms in North Carolina, USA [2]. The similar foraging rates in beneficial insect borders and Fallow borders in our study indicate the establishment of beneficial insect habitats along cropland margins may result in bobwhite population change similar to that following establishment of fallow vegetation in farm landscapes. The foraging rates of the human-imprinted bobwhite chicks in our study were greater than in similar studies (e.g., [20,23]), indicating the field borders provided the habitat conditions and abundance of available arthropods needed for chicks to consume large quantities of invertebrate prey. Fallow borders are an inexpensive, relatively simple means of providing brood habitat within farmlands, but borders planted to conserve beneficial insects appear to be a viable option for maximizing the value of set-aside lands for native arthropods, birds, and small mammals [17,24].

Chicks foraging in Mowed borders consumed large amounts of arthropods, but Mowed borders do not provide sufficient brood habitat for precocial game bird young. Brood habitat typically consists of diverse stands of vegetation with well-developed canopy structure and extensive bare ground [12]. Chicks foraging in vegetation with little overhead cover are highly susceptible to predation and, because of their small stature, dense vegetation at the ground level limits their ability to identify and capture arthropod prey [23,25]. We propose three possible explanations for why bobwhite foraging rates were high within Mowed borders even though they provided poor structural conditions: 1) arthropod prey were sufficiently abundant within Mowed borders, 2) chicks had been “trained” frequently in mowed areas prior to foraging trials, and 3) chicks consumed large, dead arthropods that had been killed by mowers on previous days. Availability of selected arthropod foods in Mowed borders and previous “training” in mowed areas may have allowed chicks to consume large quantities of prey despite vegetative qualities that typically hinder foraging efficiency. However, observers witnessed on multiple occasions chicks consuming dead, adult grasshoppers during trials in Mowed borders (C. J. Plush, *personal observation*). Therefore, foraging rates observed in Mowed borders may have been inflated because of the large amount of biomass contributed by adult grasshoppers.

Arthropod abundance does not necessarily correlate with foraging efficiency and is likely not a limiting factor for upland game bird chicks in field borders. Arthropod prey was abundant throughout all border treatment types, despite dramatic differences in vegetation composition and structure. These findings emphasize similar research suggesting that arthropod prey abundance is a less important determinant of foraging habitat quality than vegetation structure [19,23]. Similarly, insectivorous songbirds continued to forage in higher quality vegetative structure, even after arthropods were experimentally reduced [26]. Insufficient arthropod prey abundance can limit game bird young survival rates [25], but such instances are typically restricted to lands where invertebrate populations are substantially suppressed via widespread insecticide application [22]. Arthropod prey is abundant in uncultivated field margins, and focus should be directed toward promoting vegetation that facilitates chick mobility and provides protection from predators.

Our results suggest bobwhite chicks met invertebrate biomass demands through flexibility in arthropod food selection, and had the ability to adapt accordingly to temporal and spatial variation in arthropod abundance. Chicks consumed different quantities of specific arthropod taxa, but the total mass of arthropods consumed was often similar because chicks ate either few numbers of large arthropods (e.g., Lepidoptera larvae, grasshoppers) or high numbers of small arthropods (e.g., hemipterans, spiders, ants). Small arthropods are typically more abundant, but the availability of large arthropod foods may be more important because chicks spend considerably less energy searching for, capturing, and consuming their prey. Additional time spent foraging increases the risk of predation for young game bird chicks [27], and may limit the time available for loafing and resting, which is essential to feather growth and muscle development. However, many large arthropod food sources are difficult for young game birds to capture under normal circumstances (e.g., adult grasshoppers), and may be available only during brief time periods. For example, almost all of the Lepidopteran larvae biomass consumed by bobwhite chicks in Fallow field borders in 2010 was in August, and the species of Lepidoptera larvae consumed was the same in each chick (C. J. Plush, *unpublished data*). Apparently chicks were able to capitalize on a mass emergence of one species of caterpillar during this time. Large concentrations of caterpillars are highly ephemeral, and based on the low densities of caterpillars captured during arthropod sampling they are likely not a reliable food source throughout much of the brooding season.

Not all arthropod foods have equal nutritional value. Both beetles and ants are highly sclerotized, and consequently have lower digestibility compared to softer bodied arthropods such as spiders, hemipterans, and Lepidoptera larvae [28]. Therefore, the nutritional value of certain arthropods may be overestimated, because a large portion of their mass is indigestible. Bobwhite chicks consuming high quantities of highly sclerotized invertebrates may be wasting considerable energy digesting poor quality foods.

The high percentage of non-native grasses, particularly crabgrass and bermudagrass, within Fallow field borders and Flowers Only borders likely decreased their value to bobwhite young because of the greater risk of heat stress within these habitat patches. Adequate shade and thermal microclimate is an often overlooked, but important aspect of bobwhite brood cover. Bobwhite chicks are unable to thermoregulate adequately until 30 days of age, and consequently suffer high mortality rates if exposed to high temperatures even within short periods of time [29]. Both bermudagrass and crabgrass form dense stands along the ground level, and areas inundated by these plant species maintain temperatures that may exceed the heat-tolerance threshold for bobwhite young [30]; consequently, brooding hens typically avoid these areas in the wild [29]. We frequently observed chicks displaying signs of heat stress where bermudagrass and crabgrass were prevalent. However, these observations were restricted to the final minutes of foraging trials and did not appear to affect foraging efficiency during the majority of the trials (C. J. Plush, *personal observation*). 

Field borders planted as beneficial insect habitats are a useful management tool for providing habitat for young upland game birds while also promoting important arthropod communities. We recommend beneficial insect habitat seed mixes include native grasses, because they often out-compete non-native grass species that are otherwise prevalent within borders and diminish the borders’ value to young game birds and other wildlife. Native, warm-season grasses in the United States also may provide desirable nesting sites for northern bobwhite and other ground-nesting bird species [31,32] and small mammals [24]. Planted field borders should contain a diverse vegetation community and include the maximum number of species possible to benefit wildlife and arthropod communities.

## References

[1] Daniels RB, Gilliam JW (1996) Sediment and chemical load reduction by grass and riparian filters. J of the Am Soil Sci Soc 60:246–251.

[2] Riddle JD, Moorman CE, Pollock KH (2008) The importance of habitat shape and landscape context to northern bobwhite populations. J Wildl Manag 72: 1376-1382. doi:10.2193/2007-469.

[3] Olson DM, Wäckers FL (2007) Management of field margins to maximize multiple ecological services. J of Appl Ecol 44: 13-21.

[4] Heimpel GE, Jervis MA (2005) Does nectar improve biological control by parasitoids. In: Wäckers FL van Rijn PCJ Plant-provided food for carnivorous insects: a protective mutualism and its applications. Cambridge University Press, New York pp 267-304.

[5] Wäckers FL, van Rijn PCJ (2005) Food for protection: an introduction. In: Wäckers FL van Rijn PCJ Plant-provided food for carnivorous insects: a protective mutualism and its applications. Cambridge University Press, New York pp. 1-14.

[6] Landis DA, Menalled FD, Costamagna AC, Wilkinson TK (2005) Manipulating plant resources to enhance beneficial arthropods in agricultural landscapes. Weed Sci 53: 902-908. doi:10.1614/WS-04-050R1.1.

[7] Landis DA, Wratten SD, Gurr GM (2000) Habitat management to conserve natural enemies of arthropod pests in agriculture. Annu Rev Entomol 45: 175-201. doi:10.1146/annurev.ento.45.1.175. PubMed: 10761575. 10761575

[8] Forehand LM, Orr DB, Linker HM (2006) Insect communities associated with beneficial insect habitat plants in North Carolina. Environ Entomol 35: 1541-1549. Available online at: doi:10.1603/0046-225X(2006)35[1541:ICAWBI]2.0.CO.

[9] Rands MRW (1992) The conservation status and priorities for threatened partridges, francolins and quails in the world. In: Birkan M Potts GR Aebischer NJ Dowell SD Proceedings Perdix VI: first international symposium on partridges, quails and francolins, vol. 9 Gibier Falune Sauvage/Gaine anid Wildlife, pp. 493-502.

[10] Guthery FS (1997) A philosophy of habitat management for northern bobwhites. J Wildl Manag 61: 291-301. doi:10.2307/3802584.

[11] Sotherton NW (1998) Land use changes and the decline of farmland wildlife: an appraisal of the set-aside approach. Biol Conserv 83: 259-268. doi:10.1016/S0006-3207(97)00082-7.

[12] Taylor JS, Church KE, Rusch DH (1999) Microhabitat selection by nesting and brood-rearing northern bobwhite in Kansas. J Wildl Manag 63: 686-694. doi:10.2307/3802658.

[13] Jackson JR, Hurst GA, Gluesing EA (1987) Abundance and selection of invertebrates by northern bobwhite chicks. Proc of the Southeast Assoc of Fish and Wildl Agencies 41 pp. 303–310.

[14] Aebischer NJ, Ewald JA (2010) Grey partridge (*Perdix* *perdix*) in the UK: recovery status, set-aside and shooting. Ibis 152: 530-542. doi:10.1111/j.1474-919X.2010.01037.x.

[15] Sauer JR, Hines JE, Fallon J, Pardieck KL, Ziolkowski DJ Jr et al. (2011) The North American Breeding Bird Survey, results and analysis 1966 - 2010. version 12.07.2011 USGS Patuxent Wildlife Research Center, Laurel, MD.

[16] Fiedler AK, Landis DA (2007) Attractiveness of Michigan native plants to arthropod natural enemies and herbivores. Environ Entomol 36: 751-765. Available online at: doi:10.1603/0046-225X(2007)36[751:AOMNPT]2.0.CO;2. PubMed: 17716466. 17716466 10.1603/0046-225x(2007)36[751:aomnpt]2.0.co;2

[17] Plush CJ, Moorman CE, Orr DB, Reberg-Horton C (2013) Overwintering sparrow use of field borders planted as beneficial insect habitats. J Wildl Manag 77: 200-206. doi:10.1002/jwmg.436.

[18] Kimmel RO, Healy WM (1987) Imprinting: a technique for wildlife research. In: Kimmel RO Schulz JW Mitchell GJ Perdix IV: Gray Partridge Workshop. Minnesota Department of Natural Resources, Madelia, USA pp. 39-52.

[19] Palmer WE, Lane II MW, Bromley PT (2001) Human-imprinted northern bobwhite chicks and indexing arthropod foods in habitat patches. J Wildl Manag 65: 861-870. doi:10.2307/3803035.

[20] Smith MD, Burger Jr LW (2005) Use of imprinted northern bobwhite chicks to assess habitat-specific arthropod availability. Wildl Soc Bull 33: 596-605. Available online at: doi:10.2193/0091-7648(2005)33[596:UOINBC]2.0.CO;2.

[21] Harper CA, Guynn Jr DC (1998) A terrestrial vacuum sampler for macroinvertebrates. Wildl Soc Bull 26: 302-306.

[22] Palmer WE (1995) Effects of modern pesticides and farming systems on northern bobwhite quail ecology. Dissertation, North Carolina State University.

[23] Doxon ED, Carroll JP (2010) Feeding ecology of ring-necked pheasant and northern bobwhite chicks in conservation reserve program fields. J Wildl Manag 74: 249-256. doi:10.2193/2008-522.

[24] Moorman CE, Plush CJ, Orr DB, Reberg-Horton C, Gardner B (2013) Small mammal use of field borders planted as beneficial insect habitat. Wildl Soc Bull 37: 209-215. doi:10.1002/wsb.226.

[25] Potts GR (1986) Partridge: pesticides, predation and conservation. Collins, London.

[26] Champlin TB, Kilgo JC, Moorman CE (2009) Food abundance does not determine bird use of early-successional habitat. Ecol 90: 1586-1594. doi:10.1890/08-1190.1. 19569373

[27] Potts GR (1997) Cereal farming, pesticides and grey partridges. In: Pain DJ Pienkowski MW Farming and birds in Europe. Academic Press, London pp. 150-177.

[28] Evans AR, Sanson GD (2005) Biomechanical properties of insects in relation to insectivory: cuticle thickness as an indicator of insect ‘hardness’ and ‘intractablity.’. Aust J of Zool 53: 9-19. doi:10.1071/ZO04018.

[29] Forrester ND, Guthery FS, Kopp SD, Cohen WE (1998) Operative temperature reduces habitat space for northern bobwhites. J Wildl Manag 62: 1506-1511. doi:10.2307/3802017.

[30] Burkhart JK (2004) Vegetation response in field margins managed for northern bobwhite (*Colinus**virginianus*) and potential negative impacts of bermudagrass (*Cynodon**dactylon*). Thesis, University of Georgia.

[31] George RR, Farris AL, Schwartz CC, Humburg DD, Coffey JC (1979) Native prairie grass pastures as nest cover for upland birds. Wildl Soc Bull 7: 4-9.

[32] Giuliano WM, Daves SE (2002) Avian response to warm-season grass use in pasture and hayfield management. Biol Conserv 106: 1-9. doi:10.1016/S0006-3207(01)00126-4.

